# Responses of corals to chronic turbidity

**DOI:** 10.1038/s41598-020-61712-w

**Published:** 2020-03-16

**Authors:** Ross Jones, Natalie Giofre, Heidi M. Luter, Tze Loon Neoh, Rebecca Fisher, Alan Duckworth

**Affiliations:** 1 Australian Institute of Marine Science (AIMS), Townsville, QLD and Perth, WA Australia; 2 Western Australian Marine Science Institution, 35 Stirling Highway, Crawley, WA 6009 Australia

**Keywords:** Environmental impact, Ecophysiology

## Abstract

Dredging increases suspended sediment concentrations (SSCs), causing elevated water turbidity (cloudiness) and light attenuation. Close to dredging, low light periods can extend over many days, affecting phototrophic epibenthic organisms like corals. To improve the ability to predict and manage dredging impacts, we tested the response of corals to an extended period of elevated turbidity using an automated sediment dosing system that precisely controlled SSCs and adjusted light availability accordingly. Replicates of four common species of corals encompassing different morphologies were exposed to turbidity treatments of 0–100 mg L^−1^ SSC, corresponding to daily light integrals of 12.6 to 0 mol quanta m^−2^ d^−1^, over a period of ∼7 weeks. Symbiotic dinoflagellate density and algal pigment concentration, photosynthetic yields, lipid concentrations and ratios and growth varied among the turbidity treatments, with corals exhibiting photoacclimation within low turbidity treatments. A range of physiological responses were observed within the high turbidity treatments (low light), including bleaching and changes in lipid levels and ratios. Most corals, except *P. damicornis*, were capable of adjusting to a turbidity treatment involving a mean light level of 2.3 mol photons m^−2^ d^−1^ in conjunction with a SSC of 10 mg L^−1^ over the 7 week period.

## Introduction

Elevated turbidity (water cloudiness) from natural resuspension events^1–4^, terrestrial runoff^5^ and dredging activities^6–8^ is a well-known hazard to benthic marine communities^9^. Knowing when the hazard becomes a risk to sensitive organisms such as corals depends on understanding tolerance limits. For dredging, this knowledge can be used for management purposes and alert dredging proponents of water quality conditions when effects may occur allowing them to make changes to dredging schedules as required (i.e. reactive management^10^). The same understanding can be used with hydrodynamic and sediment transport models^11^ to predict the possible areal extent of impacts before dredging at the environmental impact assessment stage^12,13^.

There are several different mechanisms (cause-effect pathways) whereby the increased turbidity from suspended sediments could affect the health of the underlying communities; for recent reviews for tropical organisms see^7,14–17^. The key stressors or pressures associated with elevated turbidity include elevated suspended sediment concentrations (SSCs), sediment deposition as the sediments fall back out of suspension, and a decrease in the quantity and quality of benthic light through scattering and attenuation. These pressures can act either alone and/or in combination^7^.

Temporal and spatial changes in pressure fields associated with dredging activities have only recently been described in detail^7,8,18^. These studies of several large-scale capital dredging projects in tropical Western Australia showed that within a few hundred metres of dredging the intensity, duration and frequency of turbidity events increased by 10–, 5– and 3–fold respectively compared to baseline (pre-dredging) levels. SSCs varied 2–3 orders of magnitude over a day and although occasionally exceeding hundreds of mg L^−1^, over longer periods (i.e. weeks) the near worst case scenario (95^th^ percentile) was typically a few tens of mg L^−1^. The high turbidity levels profoundly affected underwater light levels with sites close to dredging frequently experiencing days in darkness and extended periods of very low light. The water quality data also indicated a strong power-decay relationship, with conditions improving with increasing distance from dredging^19^.

In one of these studies the *in situ* water quality measurements of SSC, benthic light availability and estimates of sediment deposition were conducted in conjunction with observations of the health of hundreds of individually marked corals at 5–10 m depth. The corals were examined at roughly fortnightly intervals over an extended (200 d) dredging period. Threshold values for the probability of non-zero mortality of shallow water corals (relative to mortality observed at control sites) over different running mean intervals were derived, suggesting strict thresholds (where the avoidance of adverse environmental impacts are prioritized) of ∼1 mol quanta m^2^ d^−1^ of photosynthetically active radiation (PAR) and SSCs of ~10 mg L^−1^ over a 14 day period. Although elevated turbidity and light reduction could be detected many kilometres away from the dredging (see^20,21^), biological effects occurred considerably closer to the dredging activities^19,22^.

Another approach to developing guidelines is by *ex situ* laboratory-(aquarium) based studies that allow careful manipulation and even isolation of individual variables and more detailed testing of cause-effect pathways. To be relevant, such studies need to use environmentally realistic exposure conditions^7^, which is now possible using water quality information described above. Using this approach, Bessell-Browne, *et al*.^23^ examined the effects of three SSCs crossed with three different light levels on a number of coral species. The study emphasized the significance of the light attenuation associated with high turbidity. Exposure to low light for extended periods resulted in the dissociation of the symbiosis i.e. bleaching, but if light levels were manipulated to compensate for the light reduction, then corals could survive extremely high SSCs (i.e. 100 mg L^−1^) over extended periods. Sediment deposition and smothering of corals was prevented in the study, which effectively suggested it was the light attenuating properties of the suspended sediments that was the primary concern for corals, rather than the suspended sediments themselves. Following from this experiment, Bessell-Browne *et al*. (2017b) tested the physiological responses of light limitation alone on corals and found the EC_10_ threshold for bleaching over 30 days was 1.2–1.9 mol photons m^−2^ d^−1^.

In this study, we examine the chronic effects of turbidity on a range of corals and morphologies using a suite of sub–lethal indicators to quantify the detailed physiological effects of six different turbidity levels over an extended (7 week) period. The exposure period was chosen as it is the typical duration of maintenance dredging activities in coastal waters of the Great Barrier Reef^24^. Although some capital dredging projects can last for several years, in practice spatial separation of excavation activities (i.e. along a channel) often means that reefs may only be exposed to plumes for shorter periods. Throughout the study we use the term turbidity to refer to the various treatments which are effectively combinations of elevated SSCs with associated light reduction. The light levels tested and the SSCs (as mg L^−1^) associated with the light level were empirically derived (see^7^), and based on a shallow water tropical reef. The study was intended to experimentally support the initial guideline values developed from *in situ* studies of^22^, but also to quantify the broader physiological consequences, survivorship and energetic status of corals, including lipid status, growth, zooxanthellae densities and chlorophyll concentrations over extended exposure to turbidity. Smothering of corals by sediment was prevented in the study, thereby ensuring the results are specific to turbidity tolerance.

## Results

**Figure 1:**
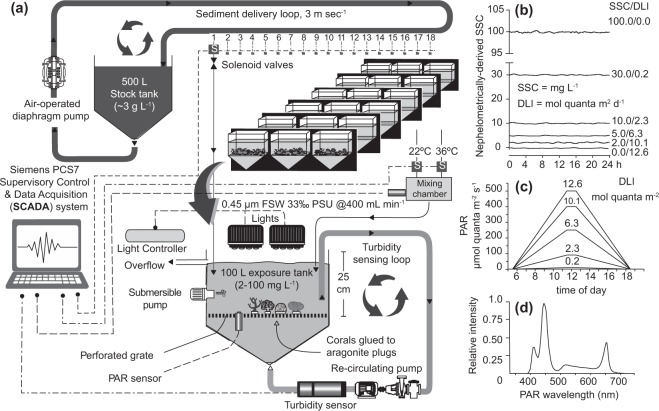
(**a**) Schematic diagram of the automated, PLC controlled sediment dosing system (see Methods text for an explanation). (**b**) Mean nephelometrically-derived SSC (mg L^−1^) recorded over a typical day in the 6 treatments (note the PLC monitored SSC every second but recorded SSC every 20 s to reduce data archiving), (**c**) PAR levels (μmol photons m^−2^ s^−1^) at 1 h intervals measured in the exposure tanks showing the ramp up and down period associated with the 13 h L:D cycle from 05:30 to 18:30. (**d**) Spectral profile of the lights used in the experiments measured using a Jaz light meter (Jaz-ULM-200, Ocean Optics, The Netherlands).

Four coral species were exposed to six turbidity treatments for 42 d in an aquarium-based study using an automated sediment dosing system regulated by a programmable logic controller (PLC, Fig. 1a). Treatments ranged from SSCs of 0, 2, 5, 10, 30 and 100 mg L^−1^ with the light levels in each treatment (expressed as a daily light integral – DLI) adjusted to approximate light levels that would occur at 5–6 m depth at the SSC, based on light profiles collected during a dredging program and described in^7^ (Fig. 1b).

### Growth and mortality

**Figure 2:**
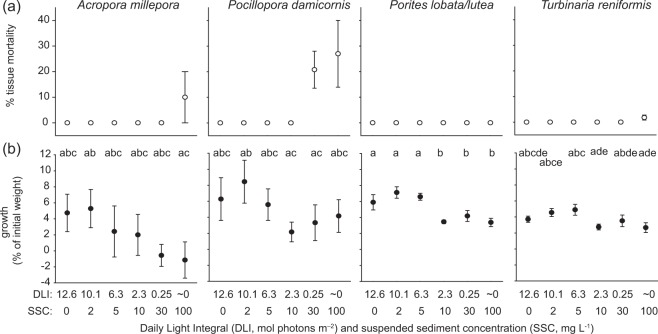
(**a**) Percent tissue mortality and (**b**) growth for the 4 coral species exposed for 42 d to SSCs ranging from 0–100 mg L^−1^ and light levels from 0–12.6 mol quanta m^−2^ d^−1^ (see Fig. 1). Error bars represent ±1 standard error and letters above plots denote significant groups according to Bayesian posterior probability contrasts (>95% probability of difference, see Supplementary Table S1).

All replicates from all species survived the 42 d exposure period, although some exhibited partial mortality (Fig. 2a). Mean percent of exposed skeleton was 10% and 2% for *A. millepora* and *T. reniformis* respectively in the highest turbidity treatment (100 mg L^−1^/~0 mol quanta m^−2^ d^−1^, Fig. 2a). For *P. damicornis* partial mortality was observed for both the 0.25 and ~0 mol quanta m^−2^ d^−1^ treatment with mean values as high as 27% (Fig. 2a). No *Porites lobata/lutea* experienced partial mortality (Fig. 2a).

Growth rate (expressed as a % of initial weight) varied somewhat among treatments across all species and was generally lowest for the higher turbidity/low light treatments (Fig. 2b). There were high model probabilities for a model containing DLI compared to an intercept only model, indicating a relatively strong effect of treatment on growth rate (all AICc ωi values > 0.9, Table 1). *A. millepora* showed a relatively consistent decline in growth rate with increasing turbidity/decreasing DLI (Fig. 2b). For *P. damicornis* growth was more variable across treatments but was highest for the three lowest turbidity treatments (highest DLI), and lower for treatments with ≤2.3 mol quanta m^−2^ d^−1^ (Fig. 2b). For *Porites lobata*/*lutea*, growth was about twice as high for corals exposed to the three lowest turbidity (highest light) treatments than the highest turbidity (lowest light) treatments with these two groups clearly delineated statistically (Fig. 2b). For *T. reniformis*, growth was highest for corals in the lowest three turbidity (highest light) treatments, peaking at 6.3 mol quanta m^−2^ d^−1^/5 mg L^−1^, although there was substantial overlap among treatments (Fig. 2b).

**Table 1:** Relative model weights for *A. millepora*, *P. damicornis*, *Porites lobata/lutea* and *T. reniformis* examining effects of 42 days of DLI and SSC treatments on zooxanthella density, pigment concentration per area (Chl *a*, *c*, and peridinin per cm^2^), dark-adapted *F*_v_/*F*_m_, total lipid concentration (percent), lipid ratio and final buoyant weight.

	*Acropora millepora*	*Pocillopora damicornis*	*Porites lobata/lutea*	*Turbinaria reniformis*
	DLI/SSC	DLI/SSC	DLI/SSC	DLI/SSC
Symbiont density	0.905	1	1	0.492
Pigment conc.	0.869	0.997	0.999	0.612
dark-adapted *F*_v_/*F*_m_	0.115	0.668	0.257	0.032
Total lipids	0.272	0.989	0.968	0.977
Lipid ratio	0.909	0.993	0.94	0.999
Buoyant weight	0.998	0.997	0.998	0.916

Percent tissue mortality was unable to be tested due to so few non-zero values. Model weights are based on AICc comparison between a model with the DLI/SSC treatment effect (i.e. turbidity) and an intercept only (null) model.

### Photosynthetic efficiency, colour, symbiont density and pigment concentrations

**Figure 3:**
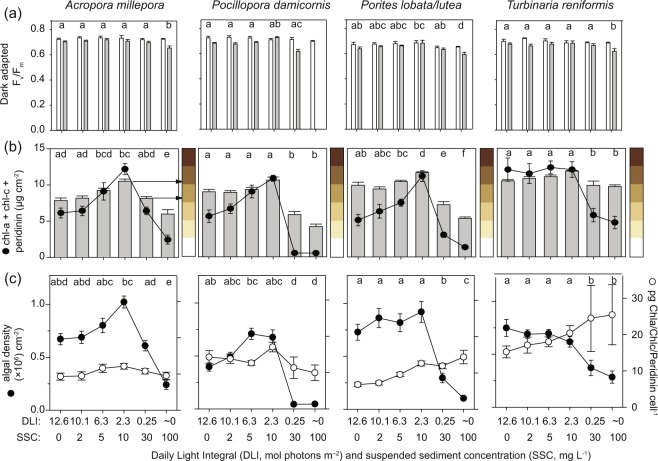
(**a**) Photochemical quantum yield (dark-adapted F_v_/F_m_) at the start of the experiment (t = 0, white bars) and end of the experiment (t = 42 d, grey bars), (**b**) Chl a and c and peridinin cm^−2^ (line plot, primary y-axis) and mean final colour (grey bars, which are associated with the E1–E6 colour squares of^25^ on the secondary y-axis), and (**c**) symbiotic dinoflagellate density (×10^6^) cm^−2^ (primary y-axis, black circles) and mean pg chl *a*, chl *c* and peridinin per symbiotic dinoflagellate (secondary y-axis, white circles), for 4 coral species exposed for 42 d to different turbidity treatments (SSCs ranging from 0–100 mg L^−1^ and light levels from 0–12.6 mol quanta m^−2^ d^−1^, see Fig. 1). Valid *F*_v_/*F*_m_ measurements For *P. damicornis* replicates exposed to 100 mg L^−1^ could not be obtained. Error bars represent ±1 standard error and letters above plots denote significant groups according to Bayesian posterior probability contrasts (>95% probability of difference, see Supplementary Table S1).

The maximum quantum yield or photochemical efficiency (*F*_v_/*F*_m_) of the algal symbionts decreased from the start to the end of the 7 week exposure period for all species (Fig. 3a). The greatest decrease occurred for the highest turbidity treatment (lowest light treatment, Fig. 3a). However, the *F*_v_/*F*_m_ values in the remnant algal population at the end of the experiment were nevertheless quite high (i.e. >0.6, Fig. 3b) and there was only limited evidence of an effect of treatment for this variable (AICc based ωi values favoured the null model, Table 1). The exception was *P. damicornis* for which there was some evidence of a treatment effect (AICc ωi = 0.668, Table 1, Fig. 3a). While valid *F*_v_/*F*_m_ measurements could not be obtained at the highest turbidity treatment, there was a high probability (>0.95%) that even the 0.25 mol quanta m^−2^ d^−1^ treatment was lower than that with the highest efficiency (Supplementary Table S1, Fig. 3a).

**Figure 4:**
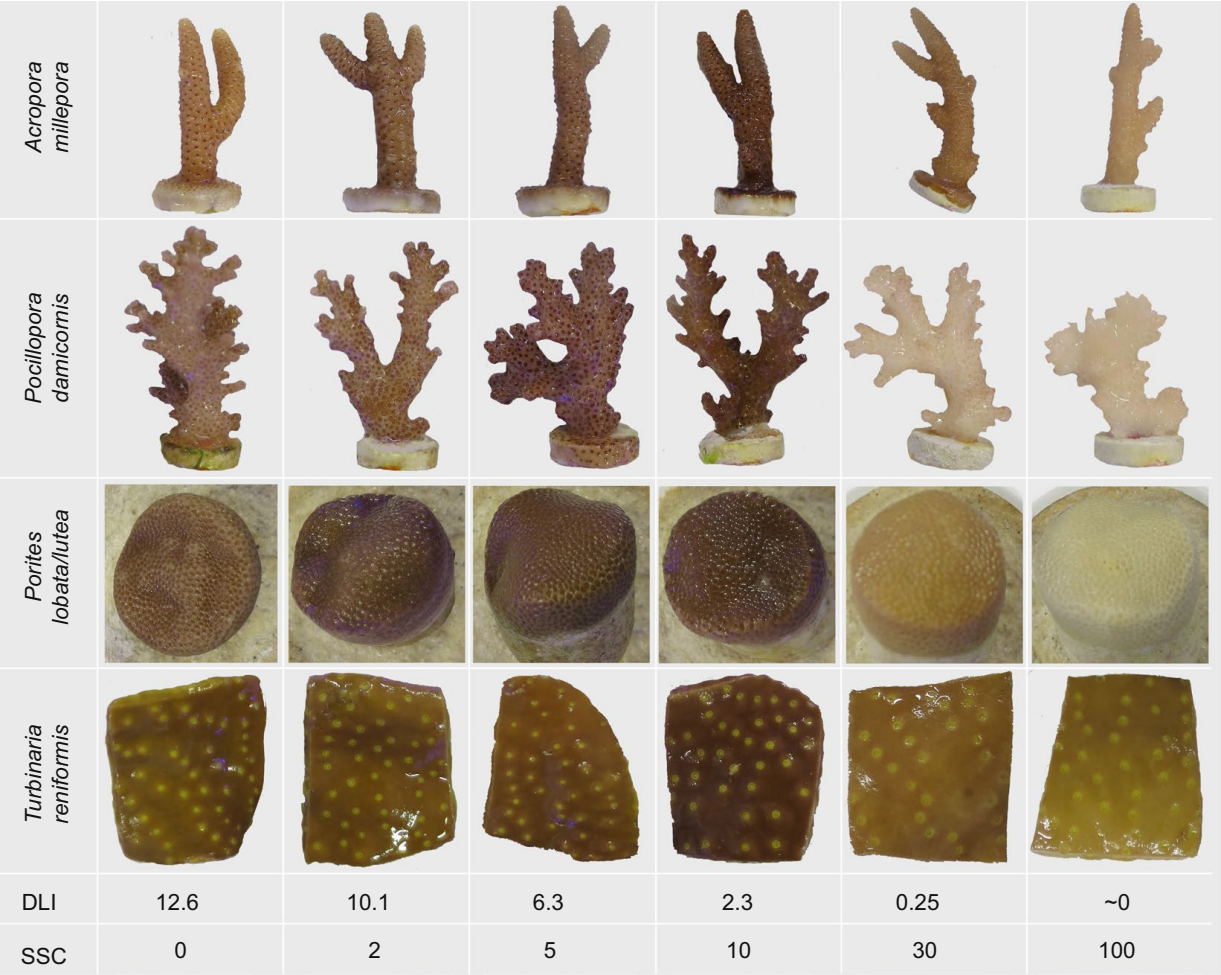
Representative fragments of *Acropora millepora*, *Pocillopora damicornis, Porites lobata/lutea* and *Turbinaria reniformis* held in 6 different turbidity treatments (daily light integral (DLI) of 12.6, 10,1, 6.3, 2.3, ∼0 mol quanta m^−2^ day^−1^ and SSCs, 0, 2, 5, 10, 30 and 100 mg L^−1^ for a period of 42 d.

There were pronounced colour differences between treatments at the end of the experiment with *A. millepora*, *P. damicornis* and *Porites lobata/lutea* appearing darker in the middle turbidity treatment (10 mg L^−1^/2.3 mol quanta m^−2^ d^−1^, Fig. 3b and 4). In the 0.25 mol quanta m^−2^ d^−1^/30 mg L^−1^ exposure, and especially the darkness exposure (~0 mol quanta m^−2^ d^−1^/100 mg L^−1^), all coral species were much lighter (i.e. bleached, Fig. 3b and 4). The bleaching occurred uniformly across the corals with no discernible preferential discoloration with respect to branch or surface orientation. Coral colour was quantified using colour reference cards^25^ and the colour changes involved an increase and decrease respectively in 2 colour categories of the reference card (Fig. 3b). There was strong evidence for a treatment effect on pigment concentrations for all species (AICc ωi > 0.612, Table 1), with the two highest SSC/lowest DLI treatments showing the lowest pigment concentrations (Fig. 3b, black circles). For the four lowest SSC treatments, for *A. millepora*, *P. damicornis* and *Porites lobata/lutea* pigment concentrations increased with increasing SSC/decreasing DLI, with highest concentrations at 2.3 mol quanta m^−2^ d^−1^ (Fig. 3b, black circles). For *T. reniformis* pigments were relatively stable at the four lowest SSC treatments (Fig. 3b, black circles).

Differences in algal symbiont density at the end of the experiment reflected the observed colour differences, with the two highest turbidity (lowest DLI) treatments having the lowest values across all species (Fig. 3c, black circles). For *A. millepora* and *Porites lobata/lutea* algal density actually increased with increasing turbidity (decreasing DLI) and was highest in the 2.3 mol quanta m^−2^ d^−1^/10 mg L^−1^ treatment (Fig. 3c, black circles). A similar pattern was observed for *P. damicornis*, although for this species highest density occurred for the 6.3 mol quanta m^−2^ d^−1^/5 mg L^−1^ treatment (Fig. 3c, black circles). For *T. reniformis* the algal density declined slightly for the four lowest turbidity treatments (0–10 mg L^−1^/12.6–2.3 mol quanta m^−2^ d^−1^, Fig. 3c, black circles) and then decreased substantially for the two highest turbidity (lowest light) treatments (Fig. 3c, black circles).

**Figure 5:**
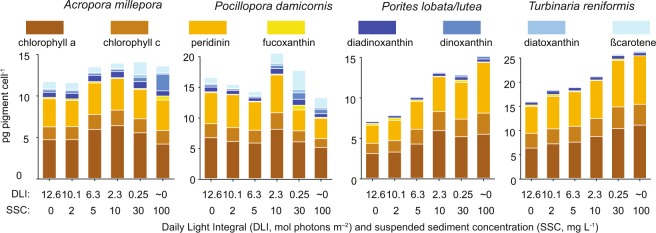
Light harvesting and xanthophyll pigment (see text) concentration per symbiotic algal cell in 4 coral species exposed for 42 d to a range of turbidity (SSCs ranging from 0–100 mg L^−1^ and corresponding light levels from 0–12.6 mol quanta m^−2^ d^−1^, see Fig. 1).

Chlorophyll *a*, chlorophyll *c*, peridinin and the xanthophylls fucoxanthin, diadinoxanthin, dinoxanthin, diatoxanthin and β-carotene were identified during the pigment analysis, with the light harvesting pigments of the chlorophyll-peridinin-protein (PCP) complex dominating all coral species (Fig. 5). Two unidentified ‘peridinin-like’ pigments were also found but were not included in the analyses due to their low concentrations. For *A. millepora* and *P. damicornis*, chl *a*, chl *c* and peridinin pigment concentration per symbiotic dinoflagellate was relatively constant with increasing turbidity (decreasing light availability), but for *Porites lobata/lutea* and *T. reniformis* the concentrations consistently increased with increasing turbidity to 2 and 1.6 × the zero turbidity (12.6 mol quanta m^−2^ d^−1^) values respectively in the highest turbidity treatment (100 mg L^−1^/0 mol quanta m^−2^ d^−1^, Fig. 3c white circles, Fig. 5).

### Lipid content

**Figure 6:**
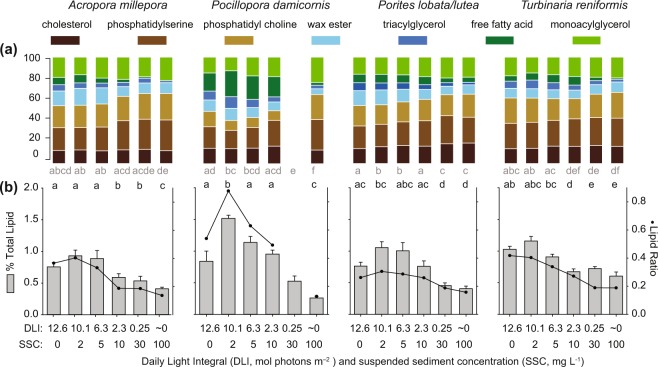
(**a**) Lipid classes and (**b**) percent total lipids (primary y-axes, grey bars) and lipid storage to structural ratio (lines and black circles, secondary y-axes))for the 4 coral species exposed for 42 d to elevated turbidity (SSCs ranging from 0–100 mg L^−1^ and light levels from 0–12.6 mol quanta m^−2^ d^−1^, see Fig. 1). Note the three structural lipid classes form the base of each bar. There was insufficient lipid content for *P. damicornis* replicates exposed to 30 mg L^−1^ to analyse for lipid classes. Error bars represent ±1 standard error and letters above plots denote significant groups according to Bayesian posterior probability contrasts (>95% probability of difference, see Supplementary Table S1), with those in grey indicating groups for total lipids, and those in black indicating groups for lipid storage to structural ratio.

Both structural lipids (cholesterol, phosphatidyl serine, and phosphatidyl) and storage lipids (wax esters, triacylglycerol, free fatty acids and monacylglycerol) were identified during the lipid analyses (Fig. 6a). Total lipid concentration varied among treatments, with high model weights for the treatment effect for *P. damicornis, Porites lobata/lutea* and *T. reniformis*, although not for *A. millepora* (Table 1). In all species, the percent total lipids increased between the zero turbidity treatment (12.6 mol quanta m^−2^ d^−1^) and 2 mg L^−1^ treatment (10.1 mol quanta m^−2^ d^−1^), and then systematically decreased with increasing turbidity (Fig. 6b, grey bars). In all cases Bayesian posterior contrasts indicated a high probability that lipids in the lowest light treatment was less than the 10.1 mol quanta m^−2^ d^−1^ treatment (Fig. 6b). The greatest overall change from highest to lowest lipid concentrations among treatments occurred for *P. damicornis* (Fig. 6b). For all species there was an effect of treatment on lipid storage to structural ratios (all AICc ωi > 0.909, Table 1), and Bayesian posterior contrasts indicated consistently lower ratios, particularly for the lowest two light treatments (Fig. 6b).

**Figure 7:**
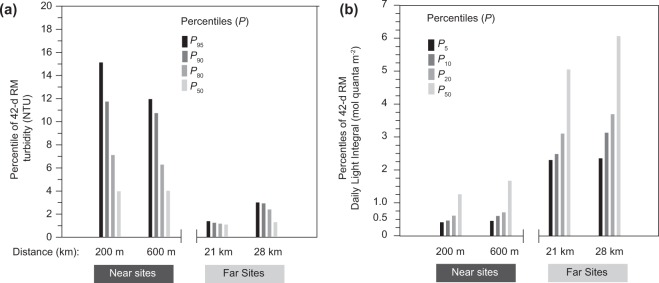
(**a**) Percentile (*P*) values (50^th^, 80^th^, 90^th^, 95^th^ for turbidity (nephelometric turbidity unit) and (**b**) *P* values for 50^th^, 20^th^, 10^th^, 5^th^ for light (daily light integral, mol quanta m^−2^) over a 42-d running mean period for sites at 7–9 depth either near to dredging (‘near sites’) or >20–30 km away (‘far sites’) from dredging during the Barrow Island dredging project^8,18,26^.

To put the 42-d laboratory based studies in context, we re-analysed turbidity and light levels collected during a major capital dredging program on a clear water reef^8,18,22^. During the dredging period, the 42-d running mean light and turbidity levels and at two sites at 7–9 m depth and few hundred metres from the dredging were calculated. The upper percentile (*P*) values^18^ were determined for turbidity and the lower percentiles determined for light. Similar analyses were conducted at reference sites 20–30 km north of the dredging at similar depths but which were not influenced by dredging-related sediment plumes^8,26^. The *P*_95_ of the sites a few hundred of metres away were 12–15 NTU, nearly an order of magnitude higher than at distantly located references >20 km away from dredging (1.5–3 NTU (Fig. 7a)). Underwater light levels mirrored this pattern, with the *P*_5_ of the 42-d running mean DLIs close to the dredging were less than 0.5 mol quanta m^−2^, compared to equivalent values at the reference sites of ∼2.3 mol quanta m^−2^ (Fig. 7b).

## Discussion

Seven weeks of exposure to a range of elevated turbidity (i.e. a combination of elevated SSCs and associated light reduction) resulted in a range of physiological and biochemical shifts in the health of corals, across all the species examined. The most conspicuous effects of the turbidity treatments were the darker pigmentation of the corals in the low to middle turbidity treatments and the lightening and bleaching of corals in the highest turbidity (near zero light) treatment. When considered collectively, and despite some differences between species and growth forms, the suite of sublethal indices suggest acclimation and tolerance in the low to mid turbidity treatments and then a transition to light deprivation at the highest turbidity (zero light) treatment. Bleaching of corals under conditions of low light involved the dissociation of the coral-algal symbiosis which is a well-known stress response of corals^27^. This transition point occurs between the 2.3 mol quanta m^2^ day^−1^ (in combination with 10 mg L^−1^) and 0.25 mol quanta m^2^ day^-^(in combination with 30 mg L^−1^) treatment and provides experimental support for guidelines derived from combined *in situ* water quality sampling and coral health monitoring on a shallow, clear water reef system during a large scale capital dredging project^22^.

The caramel brown colour of corals, in the absence of strong host pigments, epilithic algae and colour in the skeleton, is a product of both the density of the symbiotic dinoflagellates (family *Symbiodiniaceae*, LaJeunesse, *et al*.^28^) and concentration of their primary photosynthetic and accessory pigments. The majority of the pigments are associated with the peridinin-chlorophyll *a*-protein (PCP) complex^29,30^ including chlorophyll *a* (with absorption greatest at 435–440 nm and 670–680 nm), chlorophyll *c*2 (450–460 nm) and the carotenoid, peridinin (478–500 nm)^31^. For all species the deep brown pigmentation was caused by high pigment concentration per algal cell and for *A. millepora*, *P. damicornis* and *Porites lobata/lutea* by higher symbiont density. The higher algal pigment concentration is consistent with the photoacclimatory responses described previously for dinoflagellates^29,32,33^ and in symbiotic dinoflagellates in symbiosis with corals, is considered to be a mechanism to increase light absorptivity under progressive light limitation^34–39^.

The dark brown colouration was also caused by higher algal density in all species except *T. mesenterina* (see below). Some studies have reported no change in algal density under light reduction i.e.^37,38,40,41^, whereas others have clearly shown an increase in areal specific symbiont density in more turbid, lower light environments^39,42^. The discrepancy might be due to different methodologies used i.e. *in situ* sampling of colonies down light gradients versus *ex-situ* laboratory-based manipulative studies, or sampling techniques using whole colonies versus exterior branches. It could also be due to differences in experimental durations as increases in algal density takes several weeks to months^43^ as opposed to algal pigment changes that can occur within days^35,39^.

The bleaching of the tissues in the high turbidity (very low light) treatments was consistent across all species and involved the loss of the algal symbionts. Some of the corals turned bone-white although the *T. reniformis* explants still retained ~50% of their algal symbionts. Despite the heavy bleaching there was no whole colony mortality and partial mortality only occurred in the low light (high turbidity) treatments, and even then, only for *P. damicornis* and to a lesser extent *A. millepora*. The maximum quantum yield or photochemical efficiency (*F*_v_/*F*_m_) of the algal symbionts decreased within 2–4 weeks for all species at higher turbidity treatments. However, the *F*_v_/*F*_m_ values in the remnant algal population at the end of the experiment were nevertheless quite high (i.e. >0.6), even in the heavily bleached corals. These photochemical efficiency values are much higher than the rapid decreases that have been reported in previous studies when corals have been exposed to sediments^44,45^. As discussed in Bessell-Browne *et al*. (2017b), those exposures involved smothering of corals by organic rich sediments which results in rapid reduction in tissue O_2_ and pH, anoxia and hydrogen sulphide formation^46^. It follows that if experiments showing pronounced decreases in dark-adapted *F*_v_/*F*_m_ involved smothering, then inferences of ‘photo-physiological’ stress may be inappropriate if the measurements are made of algal symbionts that are trapped in a *milieu* of dead and decaying host tissue. The slight decreases in the quantum yield at very high turbidity (zero light) could have been due to unstacking and structural changes of the thylakoid membrane, leading to reduced electron transport^47,48^. Considering that the corals had been held in near darkness for 7 weeks (and at a SSC of 100 mg L^−1^), the relatively high quantum yields of the remnant algal populations is emphasized here, as opposed to the slight reductions.

Bleaching has been reported many times previously following exposure to darkness^47,49,50^, and in conjunction with the results of recent experiments^23,51^ it seems reasonable to attribute the dissociation of the symbiosis to the low light component of the elevated turbidity as opposed to the elevated suspended sediments. The bleaching of the corals to the low light was very uniform and without the variegated patterns often on upper sunlight exposed surfaces seen with elevated water temperatures^52–55^ and herbicides^56^. A reduction in translocation of algal-derived photosynthate to the host could be the cue to initiate the dissociation, as has been suggested for warm bleaching^57–59^.

The most conspicuous trend in the lipid analysis was a decrease in lipid content and change in lipid class composition with increasing turbidity. Excess fixed carbon translocated to the host from the algal symbionts^60–62^ can be quickly fixed into structural phospholipid (i.e. polar lipids and sterols) and acts as a precursor pool for storage lipids, principally as non-polar wax esters and triglycerides^63–68^. Lipids can represent up to 40−50% of the dry weight of corals^62,64^ and once photoautotrophy has been reduced under prolonged energy deficits and negative energy balances, lipids are likely to be one of the biochemical pools corals could draw on for energy^69^. Changes in lipid class composition (including polar storage and nonpolar structural lipids) can provide insights into how corals are consuming their lipid reserves^69,70^. The corals did not lose all lipid classes at similar rates, instead free fatty acid and triacylglycerol which consist of many lipid types that contribute greatly to cellular structure and integrity^71,72^ showed the greatest decreases. The lowering of the lipid ratios for all species exposed to SSC ≥ 10 mg L^−1^ (≤2.3 mol quanta m^−2^ d^−1^) is likely due to a gradual decreasing of reserves of lipids under sub-optimal light conditions. The results are consistent with previous studies examining effects of experimentally reduced light levels on lipid concentrations and branch growth^64^.

A notable secondary trend was that lipid concentrations were highest in the lowest turbidity/high light treatment (10.1 mol photons m^−2^ d^−1^ and 2 mg L^−1^ SSC) compared to the zero-turbidity treatment (12. 6 mol photons m^−2^ d^−1^ and ∼0 mg L^−1^) and higher turbidity treatments. Without follow up studies replicating the effect, it is not clear whether this trend indicates an optimal light level (10.1 mol photons m^−2^ d^−1^ versus 12.6 mol photons m^−2^ d^−1^) or possibly an interactive effect with a low level of sediment. However, it is noticeable that it was consistent in all four species and also largely reflected in the growth trends.

The pocilloporid coral *P. damicornis* was the most sensitive species to the reduced light and may prove to be a useful indicator of low light stress in coral communities during dredging. A recent analysis of field data of south-eastern Indian Ocean reefs also found a strong relationship between PAR kurtosis and corals from the genus *Pocillopora* and *Seriatopora*^73^, also suggesting some members of the family Pocilloporidae may be sensitive to light reduction. The dendrophyllid foliose coral, *T. reniformis* was the most tolerant species consistent with its reputation as a turbid water specialist^74^. *T. reniformis* and the morphologically similar *T. mesenterina*, typically dominate reefs in the inshore turbid reef zone of the central Great Barrier Reef^75,76^. Although *T. reniformis* lost significant lipid reserves in the highest turbidity treatments, they still managed to retain ~50% of their initial starting concentrations, nearly doubled the chlorophyll and peridinin pigment concentration per algal cell and maintained positive growth despite being held for 7 weeks in darkness and high SSCs.

Turbidity-generating activities like dredging contribute to a reduction in benthic light on top of natural periods of lower light levels from clouds, changes in water depth from tidal cycles and elevated turbidity from natural wind and wave induced sediment resuspension events. Although the hazard of short term (1–2 days) reduction in light from dredging was first identified in the 1970s^77^ whether it constitutes a risk has not been demonstrated. This study has shown that most of the shallow water corals used, except *P. damicornis*, were capable of adjusting to a turbidity treatment resulting in a mean light level of 2.3 mol photons m^−2^ d^−1^ over a seven-week period, and displayed clear physiological effects and in some case partial mortality at a mean daily light integral light of 0.25 mol photons m^−2^ d^−1^. These levels of light reduction have been measured *in situ* during dredging projects (Fig. 7) suggesting the hazard associated with light reduction is a risk during dredging activities, and supporting previously developed thresholds^22^. For branching coral morphologies which appear to be quite resilient to sediment smothering but susceptible to light limitation^19^, light-based monitoring using daily light integrals over different running mean time frames seems appropriate for use in reactive monitoring and for estimating impact zones at the environmental impact assessment stage.

## Methods

### Coral species

Experiments were conducted on two branching species, *Acropora millepora* (Ehrenberg 1834) and *Pocillopora damicornis* (Linnaeus 1758), a foliose species, *Turbinaria reniformis* (Bernard 1896) and the massive species, *Porites lobata* (Dana 1846) and/or *Porites lutea* (Milne Edwards & Haime, 1851). *P. lobata* and *P. lutea* are morphologically very similar and difficult to distinguish *in situ* due to their small and variable corallites (Veron 2000), and therefore a mixture of species was probably used and are referred to here as *Porites lobata/lutea*

All coral species were collected from ∼5 m depth at Davies and Broadhurst reefs which are mid-shelf reefs (~18 °S) in the central Great Barrier Reef. Fragments from up to 10 colonies each of the branching and foliose species were collected while 30 mm diameter cores of *Porites lobata/lutea* were cut from large colonies using a pneumatic drill. Corals were transported to the SeaSim aquarium facilities at the Australian Institute of Marine Science (Townsville, Queensland) where each fragment was glued onto numbered aragonite coral plugs. Corals were left to recover from the handling and preparation procedures for several weeks at 7.2 mol photons m^−2^ d^−1^ and fed using enriched *Artemia* spp.

### Experimental treatments and set-up

All experiments were conducted in a controlled environment room within the SeaSim which was maintained at 27.5–28 °C. Experiments were conducted in 18 × 115 L clear PVC tanks filled with 100 L of 0.4 µm filtered seawater, which received a continuous supply of filtered seawater pumped into each tank at rate of 400 mL min^−1^ (to ensure several water changes every day). Experiments were conducted at a water temperature of 27 °C achieved by mixing two streams of FSW (22 °C and 36 °C) through manipulating valves regulated through a Siemens PCS7 Supervisory Control and Data Acquisition (SCADA) system (see below) and with feedback from temperature thermistor in a mixing chamber (Fig. 1a). During the experiment corals (three individuals of each species per tank) were placed on a fibre reinforced plastic grating (70% open) at a depth of 25 cm and exposed to 6 different suspended sediment concentrations (SSCs, 0, 2, 5, 10, 30 and 100 mg L^−1^) each with an associated light intensity (ranging from a maximum of 0–500 µmol quanta m^−2^ s^−1^, see Fig. 1a and below) for a period of 42 d. Three tanks were used per treatment.

The sediments used were collected from Davies Reef and were biogenic and predominantly calcium carbonate based. After collection, sediments were screened to 2 mm and ground with a rod mill grinder until the mean grain size was ~30 µm (range: 3–64 µm, measured using laser diffraction techniques (Mastersizer 2000, Malvern instruments Ltd, UK)). Sediments were thus predominately silt-sized, and typical for sediments in dredge plumes^7^. Total organic content of the sediment was 0.25% (w/w).

Elevated SSCs were initially created in each tanks by dosing with small (20 mL) aliquots of a highly concentrated (≥3 g L^−1^) stock solution recirculating at high velocity (>3 m s^−1^) in the ‘sediment delivery loop’ (Fig. 1a) and delivered in short (0.5 s) pulses from a 500 L reservoir into each tank. Sediments in each tank were kept in a homogenous suspension using both a recirculating pump (Iwaki MD-70RT, Japan) and a submersible pump (VorTeck, EcoTech Marine, USA) which prevented sediment accumulation on the surface of the corals or tank bottom. Turbidity in each tank was measured in the ‘turbidity sensing loop’ using a nephelometer (Turbimax CUS31, Endress & Hauser, Germany, Fig. 1a). Conversion factors were used to relate nephelometric turbidity units (NTUs) to SSCs (mg L^−1^), based on filtering water samples through pre-weighed 0.4 µm polycarbonate filters, washing in deionized water, drying the samples (60 °C for 24 h) and then re-weighing the filters. All nephelometers were connected to the SCADA system, which recorded turbidity and episodically controlled the opening of the solenoid valves to inject sediment into each of the independent tanks from the sediment delivery loop (Fig. 1a) to replace sediment lost by the water exchanges and maintain the SSCs at the desired level. The custom Model Predictive Control logic was designed to manage potentially inconsistent aliquots (both for volume and concentration) of sediment through a self-learning process, where the system kept acquiring in real time data on the effectiveness of a train of doses, as well as the rate of sediment depletion due to settlement and dilution; this strategy gave the dosing logic both stability and the ability to rapidly reach the turbidity Set Value without overshooting. Average nephelometrically-derived SSCs were typically within 1–2 mg L^−1^ of the desired values for all treatments, shown for a typical day in Fig. 1b.

Corals were illuminated by LED aquarium lights (Hydra FiftyTwo, Aquaria Illumination, USA, see Fig. 1d for a spectral profile) suspended above each tank. The lights provided a 13 h light:dark cycle (L:D) from 05:30 to 18:30, composed a 6 h ramping up and down period and a 1 h period of maximum light intensity from 11:30 to 12:30 each day (Fig. 1c). Light intensity was controlled by positioning lights at various heights above each tank and by adjusting their power output, which was verified by measuring PAR at noon using a LI-192 Underwater Quantum sensor (LI-COR, USA). For the lowest turbidity treatment, the daily light integral (DLI; total summed PAR over the course of the day) was 12.1 mol quanta m^−2^. For the other treatments, the maximum light levels was adjusted to approximate light levels that would occur at 5–6 m depth given the suspended sediment concentration of each treatment, based on light profiles collected during a dredging program and described in^7^. Black boards were used between neighbouring tanks to prevent any light contamination (Fig. 1a). Corals were fed weekly with enriched *Artemia* spp. during the 42 d experiment^78^.

### Coral size, weight and colour

All corals were photographed at the start and end of the experiment next to a colour health monitoring chart^25^ to allow an examination of colour changes, and a scale to allow estimates of their length for *A. millepora* and *P. damicornis* and surface area for *Porites lobata/lutea* and *T. reniformis* calculated using ImageJ ImageJ, Version 1.49^79^. All corals were weighed at the start and end of the experiment using the buoyant method^80^.

To examine colour changes, the E1–E6 colour squares on the colour reference chart^25^ were used as they best reflect the colours of the 4 studied species; the colour squares range from white, representing bleaching, to dark brown. Each colour square was individually selected in ImageJ and then the histogram function (under ‘Analyze’) digitally converted each pixel to grayscale. For each coral, the polygon selection tool in ImageJ was used to draw around the replicate, digitised to grayscale to calculate a colour mean, which was then compared to the means of the colour squares. Initial coral colour was statistically similar (ANOVA: P > 0.05) between SSC for all species.

### Chlorophyll fluorometry

Dark-adapted (for ≥1 h), quantum yield (as the ratio of variable fluorescence *F*_v_ to maximal fluorescence F_m_ of the symbiotic dinoflagellates) was measured at the start and end of the experiments using a Mini-PAM fluorometer (Walz GmbH, Germany).

### Lipids, symbiotic dinoflagellate and pigments

At the end of the experiment, corals were flash frozen in liquid nitrogen and stored at −80 °C. Tissues were removed from the skeleton using air blasting in 20 mL of 0.22 µm filtered seawater. The slurry was homogenised for 30 s, the volume recorded, and then aliquots were taken for lipid (10 mL), symbiotic dinoflagellate density (1 mL and fixed in 10% buffered formalin), and pigment (1 mL) analyses. The lipid aliquots were temporarily stored at −20 °C, while the other two samples were stored at −80 °C. The surface area of the corals was determined using the wax dipping technique^81^.

For symbiotic dinoflagellate density, each aliquot was counted 8 times using a Neubauer haemocytometer containing 8 µL of homogenised solution. For pigment extraction each sample was centrifuged at 1500 × g for 1 min at 4 °C to obtain an algal pellet, which was then suspended in 0.9 mL of pre-chilled buffered extraction solvent (methanol-2.8 mM tetrabutylammonium acetate pH 6.5; 98:2), sonicated for 10 s and stored in the dark on ice for 30 mins. The sample was centrifuged again, with the supernatant subsequently transferred to a volumetric flask. This extraction process was repeated, with the combined supernatant made up with the extraction solvent to the prescribed volume and then passed through a 0.2 µm filter before chromatography. All samples and extracts were kept on ice throughout the extraction process which was performed under dimmed light. The extracts were analysed by chromatography on a Waters Acquity UPLC (Waters, USA) system coupled to photo-diode array (PDA) detector. The extracted pigments were separated on an Acquity UPLC BEH C8 column (2.1 × 150 mm; 1.7 µm) over a 20 min run using a binary gradient of methanol-tetrabutylammonium acetate (70:30) and methanol-acetonitrile (50:50) at a constant flow rate of 0.45 mL min^−1^ and a column temperature of 60 °C. Finally, the various pigments in each sample were identified by retention time and PDA spectral confirmation before being quantified against calibration curves that were established under the same running conditions using certified reference pigments (DHI, Denmark).

For lipid analysis, each sample was first freeze-dried for 48 h, placed in a 10 mL test tube together with 2 mL of dichloromethane-methanol (2:1). The sample was sonicated for 10 min, extracted 3 times with 7 mL (total) of dichloromethane-methanol using a solvent rinsed cotton-stuffed Pasteur pipette, washed with 3.5 mL of 0.44% KCl in methanol-H_2_O (3:1) and left to separate overnight. The next day, the bottom lipid layer was carefully removed using a glass syringe, placed into a pre-weighed vial, dried-down under nitrogen, and finally weighed. For samples with sufficient lipid content (>1 mg), the amount of storage lipids (wax ester, triacylglycerol, free fatty acid, monoacylglycerol) and structural lipids (cholesterol, phosphatidylserine, phosphatidylcholine) were extracted following^82^, and analysed using a thin layer chromatography-flame ionization detector (Mitsubishi Chemical Medience, Japan). The lipid ratio of storage to structural lipids was then calculated according to^82,83^.

### Data analysis

A generalised linear mixed modelling (GLMM) framework was used to assess the strength of the treatment effect and examine the differences among individual treatments. GLMMs were fit individually for each species, for each of the response variables of interest. For growth (buoyant weight expressed as % of initial weight) we used a Gaussian distribution, as this can theoretically take positive and negative values (corals can both grow and shrink) and appeared to be normally distributed. For the remaining variables (including zooxanthella density (algal density), pigment concentration (µg chl *a*, chl *c* and peridinin cm^−2^), photochemical quantum yield (dark-adapted F_v_/F_m_), total lipids, and storage to structural lipid ratio) a Gamma distribution was used as these were continuous on the scale of >0. Models were fit using the glmer function in the R package lme4^84^ using tank as a random effect. Relative model weights^85^ were calculated using AICc values for the model including the treatment effect, and a null model including only the random tank effect. We used a Bayesian approach for comparing individual treatment effects for each species, based on posterior contrasts. Bayesian models of the equivalent GLMM design were fit using an integrated nested Laplace approximation (INLA) for approximating posterior distributions (see Rue, *et al*.^86^). Statistically similar groupings were based on a posterior probability of similarity of 0.95% or greater.

## Supplementary information

Supplementary Information.

Supplementary Information2.
