# Supplementary material for: Responses of corals to chronic turbidity
Source: Sci Rep. 2020 Mar 16;10:4762. doi: 10.1038/s41598-020-61712-w (PMC7075922; doi:10.1038/s41598-020-61712-w)
Supplement: Supplementary file 1 — Supplementary Information. [file 41598_2020_61712_MOESM1_ESM.docx]

**Supplementary Table S1.** Probability contrasts between DLI treatments identified for each response variable and species.
