# Supplementary material for: Responses of corals to chronic turbidity
Source: Sci Rep. 2020 Mar 16;10:4762. doi: 10.1038/s41598-020-61712-w (PMC7075922; doi:10.1038/s41598-020-61712-w)
Supplement: Supplementary file 2 — Supplementary Information2. [file 41598_2020_61712_MOESM2_ESM.docx]

|  |  | Buoyant weight | | | | | | Pigment concentration | | | | | | Lipid ratio | | | | | |
| --- | --- | --- | --- | --- | --- | --- | --- | --- | --- | --- | --- | --- | --- | --- | --- | --- | --- | --- | --- |
|  | DLI | 12.6 | 10.1 | 6.3 | 2.3 | 0.25 | ~0 | 12.6 | 10.1 | 6.3 | 2.3 | 0.25 | ~0 | 12.6 | 10.1 | 6.3 | 2.3 | 0.25 | ~0 |
|  | 12.6 | 1 | 0.5 | 0.23 | 0.2 | 0.03 | 0.02 | 1 | 0.4 | 0.04 | 0 | 0.42 | 0 | 1 | 0.25 | 0.29 | 0 | 0 | 0 |
|  | 10.1 | 0.5 | 1 | 0.23 | 0.18 | 0.04 | 0.03 | 0.4 | 1 | 0.02 | 0 | 0.47 | 0 | 0.25 | 1 | 0.09 | 0 | 0 | 0 |
|  | 6.3 | 0.23 | 0.23 | 1 | 0.42 | 0.18 | 0.08 | 0.04 | 0.02 | 1 | 0.1 | 0.04 | 0 | 0.29 | 0.09 | 1 | 0 | 0 | 0 |
|  | 2.3 | 0.2 | 0.18 | 0.42 | 1 | 0.29 | 0.17 | 0 | 0 | 0.1 | 1 | 0 | 0 | 0 | 0 | 0 | 1 | 0.48 | 0.04 |
|  | 0.25 | 0.03 | 0.04 | 0.18 | 0.29 | 1 | 0.38 | 0.42 | 0.47 | 0.04 | 0 | 1 | 0 | 0 | 0 | 0 | 0.48 | 1 | 0.04 |
|  | ~0 | 0.02 | 0.03 | 0.08 | 0.17 | 0.38 | 1 | 0 | 0 | 0 | 0 | 0 | 1 | 0 | 0 | 0 | 0.04 | 0.04 | 1 |
|  | 12.6 | 1 | 0.28 | 0.4 | 0.08 | 0.17 | 0.26 | 1 | 0.25 | 0.08 | 0.05 | 0 | 0 | 1 | 0 | 0.13 | 0.22 |  | 0 |
|  | 10.1 | 0.28 | 1 | 0.16 | 0.01 | 0.07 | 0.09 | 0.25 | 1 | 0.26 | 0.08 | 0 | 0 | 0 | 1 | 0.01 | 0 |  | 0 |
|  | 6.3 | 0.4 | 0.16 | 1 | 0.12 | 0.25 | 0.35 | 0.08 | 0.26 | 1 | 0.3 | 0 | 0 | 0.13 | 0.01 | 1 | 0.07 |  | 0 |
|  | 2.3 | 0.08 | 0.01 | 0.12 | 1 | 0.37 | 0.2 | 0.05 | 0.08 | 0.3 | 1 | 0 | 0 | 0.22 | 0 | 0.07 | 1 |  | 0 |
|  | 0.25 | 0.17 | 0.07 | 0.25 | 0.37 | 1 | 0.37 | 0 | 0 | 0 | 0 | 1 | 0.43 |  |  |  |  |  |  |
|  | ~0 | 0.26 | 0.09 | 0.35 | 0.2 | 0.37 | 1 | 0 | 0 | 0 | 0 | 0.43 | 1 | 0 | 0 | 0 | 0 |  | 1 |
|  | 12.6 | 1 | 0.08 | 0.24 | 0 | 0.03 | 0 | 1 | 0.12 | 0.04 | 0 | 0 | 0 | 1 | 0 | 0.12 | 0.38 | 0.01 | 0 |
|  | 10.1 | 0.08 | 1 | 0.38 | 0 | 0.01 | 0 | 0.12 | 1 | 0.15 | 0 | 0 | 0 | 0 | 1 | 0.21 | 0 | 0 | 0 |
|  | 6.3 | 0.24 | 0.38 | 1 | 0 | 0.02 | 0 | 0.04 | 0.15 | 1 | 0.03 | 0 | 0 | 0.12 | 0.21 | 1 | 0.1 | 0 | 0 |
|  | 2.3 | 0 | 0 | 0 | 1 | 0.18 | 0.42 | 0 | 0 | 0.03 | 1 | 0 | 0 | 0.38 | 0 | 0.1 | 1 | 0.01 | 0 |
|  | 0.25 | 0.03 | 0.01 | 0.02 | 0.18 | 1 | 0.15 | 0 | 0 | 0 | 0 | 1 | 0 | 0.01 | 0 | 0 | 0.01 | 1 | 0.08 |
|  | ~0 | 0 | 0 | 0 | 0.42 | 0.15 | 1 | 0 | 0 | 0 | 0 | 0 | 1 | 0 | 0 | 0 | 0 | 0.08 | 1 |
|  | 12.6 | 1 | 0.09 | 0.1 | 0.12 | 0.43 | 0.1 | 1 | 0.42 | 0.49 | 0.49 | 0 | 0 | 1 | 0.41 | 0.04 | 0 | 0 | 0 |
|  | 10.1 | 0.09 | 1 | 0.37 | 0 | 0.07 | 0 | 0.42 | 1 | 0.42 | 0.39 | 0 | 0 | 0.41 | 1 | 0.11 | 0 | 0 | 0 |
|  | 6.3 | 0.1 | 0.37 | 1 | 0.01 | 0.04 | 0 | 0.49 | 0.42 | 1 | 0.46 | 0 | 0 | 0.04 | 0.11 | 1 | 0.03 | 0 | 0 |
|  | 2.3 | 0.12 | 0 | 0.01 | 1 | 0.17 | 0.47 | 0.49 | 0.39 | 0.46 | 1 | 0 | 0 | 0 | 0 | 0.03 | 1 | 0 | 0 |
|  | 0.25 | 0.43 | 0.07 | 0.04 | 0.17 | 1 | 0.15 | 0 | 0 | 0 | 0 | 1 | 0.14 | 0 | 0 | 0 | 0 | 1 | 0.47 |
|  | ~0 | 0.1 | 0 | 0 | 0.47 | 0.15 | 1 | 0 | 0 | 0 | 0 | 0.14 | 1 | 0 | 0 | 0 | 0 | 0.47 | 1 |
|  |  | Total lipids | | | | | | Dark-adapted Fv/Fm | | | | | | Zooxanthellae density | | | | | |
|  | 12.6 | 1 | 0.06 | 0.19 | 0.05 | 0.02 | 0 | 1 | 0.27 | 0.2 | 0.33 | 0.36 | 0 | 1 | 0.44 | 0.17 | 0 | 0.27 | 0 |
|  | 10.1 | 0.06 | 1 | 0.3 | 0 | 0 | 0 | 0.27 | 1 | 0.33 | 0.47 | 0.21 | 0 | 0.44 | 1 | 0.19 | 0 | 0.24 | 0 |
|  | 6.3 | 0.19 | 0.3 | 1 | 0.01 | 0 | 0 | 0.2 | 0.33 | 1 | 0.35 | 0.15 | 0 | 0.17 | 0.19 | 1 | 0.04 | 0.08 | 0 |
|  | 2.3 | 0.05 | 0 | 0.01 | 1 | 0.25 | 0.01 | 0.33 | 0.47 | 0.35 | 1 | 0.25 | 0 | 0 | 0 | 0.04 | 1 | 0 | 0 |
|  | 0.25 | 0.02 | 0 | 0 | 0.25 | 1 | 0.14 | 0.36 | 0.21 | 0.15 | 0.25 | 1 | 0.01 | 0.27 | 0.24 | 0.08 | 0 | 1 | 0 |
|  | ~0 | 0 | 0 | 0 | 0.01 | 0.14 | 1 | 0 | 0 | 0 | 0 | 0.01 |  | 0 | 0 | 0 | 0 | 0 | 1 |
|  | 12.6 | 1 | 0 | 0.02 | 0.23 | 0.01 | 0 | 1 | 0.05 | 0.22 | 0 | 0 |  | 1 | 0.26 | 0.06 | 0.07 | 0 | 0 |
|  | 10.1 | 0 | 1 | 0.02 | 0 | 0 | 0 | 0.05 | 1 | 0.03 | 0 | 0.04 |  | 0.26 | 1 | 0.14 | 0.12 | 0 | 0 |
|  | 6.3 | 0.02 | 0.02 | 1 | 0.08 | 0 | 0 | 0.22 | 0.03 | 1 | 0 | 0.01 |  | 0.06 | 0.14 | 1 | 0.48 | 0 | 0 |
|  | 2.3 | 0.23 | 0 | 0.08 | 1 | 0 | 0 | 0 | 0 | 0 | 1 | 0 |  | 0.07 | 0.12 | 0.48 | 1 | 0 | 0 |
|  | 0.25 | 0.01 | 0 | 0 | 0 | 1 | 0 | 0 | 0.04 | 0.01 | 0 | 1 |  | 0 | 0 | 0 | 0 | 1 | 0.41 |
|  | ~0 | 0 | 0 | 0 | 0 | 0 | 1 |  |  |  |  |  |  | 0 | 0 | 0 | 0 | 0.41 | 1 |
|  | 12.6 | 1 | 0.01 | 0.02 | 0.47 | 0 | 0 | 1 | 0.16 | 0.12 | 0.02 | 0.43 | 0.01 | 1 | 0.18 | 0.32 | 0.16 | 0 | 0 |
|  | 10.1 | 0.01 | 1 | 0.31 | 0 | 0 | 0 | 0.16 | 1 | 0.44 | 0.15 | 0.12 | 0 | 0.18 | 1 | 0.39 | 0.41 | 0 | 0 |
|  | 6.3 | 0.02 | 0.31 | 1 | 0 | 0 | 0 | 0.12 | 0.44 | 1 | 0.15 | 0.06 | 0 | 0.32 | 0.39 | 1 | 0.31 | 0 | 0 |
|  | 2.3 | 0.47 | 0 | 0 | 1 | 0 | 0 | 0.02 | 0.15 | 0.15 | 1 | 0 | 0 | 0.16 | 0.41 | 0.31 | 1 | 0 | 0 |
|  | 0.25 | 0 | 0 | 0 | 0 | 1 | 0.25 | 0.43 | 0.12 | 0.06 | 0 | 1 | 0.05 | 0 | 0 | 0 | 0 | 1 | 0 |
|  | ~0 | 0 | 0 | 0 | 0 | 0.25 | 1 | 0.01 | 0 | 0 | 0 | 0.05 | 1 | 0 | 0 | 0 | 0 | 0 | 1 |
|  | 12.6 | 1 | 0.04 | 0.07 | 0 | 0 | 0 | 1 | 0.29 | 0.45 | 0.33 | 0.34 | 0.01 | 1 | 0.35 | 0.27 | 0.16 | 0 | 0 |
|  | 10.1 | 0.04 | 1 | 0.02 | 0 | 0 | 0 | 0.29 | 1 | 0.24 | 0.15 | 0.36 | 0.01 | 0.35 | 1 | 0.48 | 0.25 | 0 | 0 |
|  | 6.3 | 0.07 | 0.02 | 1 | 0 | 0 | 0 | 0.45 | 0.24 | 1 | 0.35 | 0.32 | 0.01 | 0.27 | 0.48 | 1 | 0.25 | 0 | 0 |
|  | 2.3 | 0 | 0 | 0 | 1 | 0.23 | 0.13 | 0.33 | 0.15 | 0.35 | 1 | 0.19 | 0 | 0.16 | 0.25 | 0.25 | 1 | 0.01 | 0 |
|  | 0.25 | 0 | 0 | 0 | 0.23 | 1 | 0.05 | 0.34 | 0.36 | 0.32 | 0.19 | 1 | 0.01 | 0 | 0 | 0 | 0.01 | 1 | 0.12 |
|  | ~0 | 0 | 0 | 0 | 0.13 | 0.05 | 1 | 0.01 | 0.01 | 0.01 | 0 | 0.01 | 1 | 0 | 0 | 0 | 0 | 0.12 | 1 |
